# Position paper of diagnosis and treatment of post-extubation laryngitis in a multidisciplinary expert-based opinion

**DOI:** 10.1016/j.bjorl.2024.101401

**Published:** 2024-02-22

**Authors:** Débora Bressan Pazinatto, Rebecca Maunsell, Melissa Ameloti Gomes Avelino, Jose Faibes Lubianca Neto, Cláudia Schweiger, Jamil Pedro de Siqueira Caldas, Marcelo Barciela Brandão, Paula Pires de Souza, Fernanda Aparecida de Oliveira Peixoto, Claudia Pires Ricachinevsky, Rita C. Silveira, Cinara Andreolio, Carolina Sponchiado Miura, Daniele da Silva Jordan Volpe, Walusa Assad Gonçalves Ferri, Fabiano Bleggi Gavazzoni, Paulo Ramos David João, Silmara Aparecida Possas, Carlos Takahiro Chone

**Affiliations:** a Universidade Estadual de Campinas (UNICAMP), Hospital de Clínicas, Campinas, SP, Brazil; b Universidade Federal de Goiás, Hospital das Clínicas, Goiânia, GO, Brazil; c Hospital da Criança Santo Antônio, Porto Alegre, RS, Brazil; d Hospital de Clínicas de Porto Alegre, Porto Alegre, RS, Brazil; e Universidade Estadual de Campinas (UNICAMP), Hospital da Mulher Prof. Dr. J. A. Pinotti-Caism, Campinas, SP, Brazil; f Faculdade de Medicina de Ribeirão Preto, Hospital das Clínicas, Ribeirão Preto, SP, Brazil; g Hospital Pequeno Príncipe, Curitiba, PR, Brazil

**Keywords:** Acute lesions, Intubation, Larynx, Subglottic stenosis, Pediatrics

## Abstract

**Objectives:**

To make recommendations on the diagnosis and treatment of post-extubation laryngitis (PEL) in children with or without other comorbidities.

**Methods:**

A three-iterative modified Delphi method was applied. Specialists were recruited representing pediatric otolaryngologists, pediatric and neonatal intensivists. Questions and statements approached topics encompassing definition, diagnosis, endoscopic airway evaluation, risk factors, comorbidities, management, and follow-up. A consensus was defined as a supermajority >70%.

**Results:**

Stridor was considered the most frequent symptom and airway endoscopy was recommended for definitive diagnosis. Gastroesophageal reflux and previous history of intubation were considered risk factors. Specific length of intubation did not achieve a consensus as a risk factor. Systemic corticosteroids should be part of the medical treatment and dexamethasone was the drug of choice. No consensus was achieved regarding dosage of corticosteroids, although endoscopic findings help defining dosage and length of treatment. Non-invasive ventilation, laryngeal rest, and use of comfort sedation scales were recommended. Indications for microlaryngoscopy and bronchoscopy under anesthesia were symptoms progression or failure to improve after the first 72-h of medical treatment post-extubation, after two failed extubations, and/or suspicion of severe lesions on flexible fiberoptic laryngoscopy.

**Conclusions:**

Management of post-extubation laryngitis is challenging and can be facilitated by a multidisciplinary approach. Airway endoscopy is mandatory and impacts decision-making, although there is no consensus regarding dosage and length of treatment.

## Highlights

• Stridor is the predominant symptom of post-extubation laryngitis.
• Airway endoscopy is needed for a definitive diagnosis.
• Reflux and previous history of intubation were identified as risk factors.
• Systemic corticosteroids should be part of the medical treatment.
• Multidisciplinary approach is advised for post-extubation laryngitis management.

## Introduction

Acute inflammatory laryngeal lesions caused by endotracheal intubation can cause respiratory symptoms hours or a few days after extubation and are associated with increased morbidity including prolonged hospital stay, risks of reintubation, prolonged use of sedation, airway trauma, nosocomial infection, and development of laryngeal stenosis.^1^ Laryngeal Stenosis (LS) is a well-recognized clinical entity since 1965, when the use of prolonged endotracheal intubation began to be used in children who needed longer ventilatory support.^2^ In a recently published prospective study, the overall incidence of post-intubation subglottic stenosis (SGS) was estimated to be as high as 11%.^3^

Management of post-extubation acute laryngeal lesions is complex and needs the work of a multidisciplinary team, which includes an otolaryngologist who can diagnose and evaluate these lesions endoscopically. Unfortunately, airway evaluation and a teamwork approach between pediatric intensivists and otolaryngologists are not always widely available in our country.

The timely detection and treatment of these lesions can prevent their progression to a chronic state, thereby averting the necessity for complex open airway surgery and prolonged reliance on tracheostomy.^4^ However, so far, there is a lack of robust randomized, controlled studies defining the best treatment for the acute lesions. Randomized control studies are particularly challenging in this field due to ethical issues. Therefore, each center manages its cases according to its own expertise, reports on case series, and follows the recommendations of large patient volume airway centers. This study aims to provide recommendations on diagnosis and treatment of post-intubation acute laryngeal lesions.

## Methods

A panel of 17 physicians representing Pediatric Otolaryngology (ENT), Pediatric Intensive Care Unit (PICU) and Neonatal Intensive Care Unit (NICU) were invited via email to participate after filling in a consent form. Criteria for participation was being part of a multidisciplinary team with extensive experience in management of pediatric airway and identified as training centers for pediatric otolaryngologists by the Brazilian Academy of Pediatric Otolaryngology (ABOPe) database.

The expert panel consisted of 6 otolaryngologists, 5 pediatric intensivists and 6 neonatologists from 6 different healthcare facilities. Four hospitals were tertiary healthcare facilities and two were secondary care facilities. The minimum time of experience as a multidisciplinary team was three years.

Two experts and one study coordinator were responsible for the formulation of a three-interactive Delphi method questionnaire.

Topics included in the questionnaires were: definition of post-extubation laryngitis (PEL); symptoms of PEL; ENT evaluation; risk factors and comorbidities; diagnostic procedures; medical treatment; surgical treatment; criteria for discharge from intensive care unit and ward; follow-up.

Consensus statements were developed using a modified Delphi method, a systematic approach to achieving consensus among a panel of experts. The survey was distributed to 17 chosen specialists and responses were collected. The responders were given the opportunity to comment on the content and the format of the survey, which was modified for the second round. Only the study coordinator was not blinded to members’ identities.

Questions regarding endoscopic procedures and surgical treatment were directed only to the otolaryngologists. Questions regarding discharge from PICU and NICU were directed only to pediatricians.

To reflect the variability in practice patterns presented among experts in the field, the degree of consensus was quantified by presenting the percentage of authors who agreed with each statement. Consensus was established as a 70% agreement.

## Results

Half of the experts reported treating more than three cases of PEL per month, and over 90% treated at least one case per month. Only one neonatologist specialist stated that cases of PEL occurred less than once a month.

Among the 11 pediatricians, five work in hospitals without a designated otolaryngologist or airway team, and they find this situation troublesome.

The first questionnaire was composed of 69 both multiple choice and open questions (19), the second was reduced to 43 questions (8 open questions) and a third consisted of 4 questions. Results of the survey are summarized in Table 1.

**Table 1:** Results of the survey with statements that reached or did not reach over 70% consensus.

Statements	Consensus (%)		Answers	
	Yes	No	ENT	PED
**Definition**				
Definition of PEL: inflammation of the larynx associated to tracheal intubation that manifests as stridor and/or dysphonia and/or effortful breathing beginning hours or days following extubation associated or not to failed extubation	X (100%)		X	X
Most appropriate terminology: post extubation laryngitis	X (76.5%)		X	X
**Symptoms**				
Stridor is the most important symptom	X (70.6%)		X	X
Dysphonia and retractions were the second and third most common symptoms		X (58%)	X	X
24 h–48 h is the maximum expectation time for spontaneous resolution of symptoms before demanding endoscopic evaluation	X (70.5%)		X	X
**Diagnosis**				
Diagnosis must be confirmed by endoscopic examination	X (82.4%)		X	X
FNL is the first endoscopy procedure that should be performed in patients with PEL	X (100%)		X	X
3.2 or 3.4 mm FNL are the most used for bedside examination	X (100%)		X	X
FNL as a bedside examination		X (66.7%)	X	X
Sedation is not needed for bedside FNL	X (83.3%)		X	X
Use of topical anesthetics for bedside FNL		X (50%)	X	X
The main limitation of FNL is the evaluation of the subglottis	X (100%)		X	X
FNL allows evaluation of severe acquired laryngeal lesion (cricoid exposure, granulation tissue, subglottic stenosis)		X (66%)	X	X
**Risk factors and precautions**				
Specific length of intubation in PICU		X (60%)	X	X
Specific length of intubation in the NICU		X (68%)	X	X
Previous intubation history is considered a risk factor for PEL	X (88.2%)		X	X
GERD is considered a risk factor for PEL	X (70.6%)		X	X
Neuropathy as a condition influencing the occurrence of PEL	X (76.5%)		X	X
Cardiopathy, post-operative cardiac surgery, and sepsis as conditions influencing the occurrence of PEL		X (61.7%)	X	X
Comfort scale is considered important	X (81.8%)		X	X
Care should be taken particularly during endotracheal aspiration	X (100%)		X	X
**Clinical treatment**				
Systemic corticosteroids are part of the clinical treatment if no contra indication	X (76.5%)		X	X
Dexamethasone is the most used systemic corticosteroid	X (70.5%)		X	X
Minimal clinical treatment with systemic corticosteroid is 48 h	X (94.1%)		X	X
Maximum clinical treatment with maximum dose of systemic corticosteroid is 72 h		X (47%)	X	X
Minimum dose of dexamethasone		X (58.8%)	X	X
Maximum dose of dexamethasone		X (23.5%)	X	X
The endoscopic aspect of the lesions, described by the otolaryngologist, influences the choice of dose and duration of corticosteroid therapy	X (94.1%)		X	X
Proton pump inhibitors are part of the clinical treatment	X (70.6%)		X	X
PPI dosage varies from 1 to 2 mg/kg		X (52.9%)	X	X
Nebulized adrenaline is part of clinical treatment	X (94.1%)		X	X
Use of non-invasive ventilation is beneficial and useful	X (100%)		X	X
Inadequate sedation levels, inappropriate masks, craniofacial deformities, nasal lesions, and inexperienced physiotherapy teams are considered the causes for NIV failure	X (94,1%)		X	X
High flow nasal cannula is a useful tool in treating PEL	X (70.6%)		X	X
**Endoscopic treatment**				
Indications for MLB: following two failed extubations, severe acute laryngeals lesions seen on FNL and/or persistent stridor and dysphonia 72 h after extubation and medical treatment	X (100%)		X	X
Circumferential mucosal ulceration, ulceration with exposed cartilage, SGS and interarytenoid lesions are considered severe aspects of PEL	X (100%)		X	X
Intra or perilesional infiltration of triamcinolone is used for severe lesions	X (100%)		X	X
Airway calibration with endotracheal tube is routine during MLB	X (100%)		X	X
When dilation is indicated for acute SGS balloon is the instrument of choice	X (100%)		X	X
Mytomicin C is not used	X (100%)		X	X
After MLB intubated patients will be extubated 24–72 h	X (100%)		X	X
A smaller uncuffed tube is the choice following MLB	X (83.3%)		X	X
Laryngeal rest and appropriate sedation until extubation are recommended	X (100%)		X	X
Corticosteroid and antibiotic ointment applied around the tube is beneficial in treatment of PEL	X (100%)		X	X
Systemic corticosteroids and PPI are recommended following MLB for PEL	X (94.1%/70.6%)		X	X
Tracheal secretion culture is not part of the routine	X (94.1%)		X	X
Tracheal secretion cultures are indicated when secondary infection is suspected, in cases of poor outcome and/or severe lesions are encountered in endoscopy	X (93.7%)		X	X
**Follow-up**				
The ENT surgeon is responsible for tracheostomy indication	X (88.2%)		X	X
The ENT surgeon should be involved in the tracheostomy follow-up	X (100%)		X	X
Discharge from the PICU and NICU is a pediatric decision. ENT reevaluation is demanded only if stridor or dysphonia	X (70%)		X	X
PEL patients should have peripheral venous access during entire ICU and ward stay while symptomatic	X (76.4%)		X	X
Discharge from hospital only after ENT evaluation	X (75%)		X	X
ENT reevaluation in outpatient setting after 1‒3 weeks regardless of symptoms	X (83.3%)		X	X
Need of MLB in follow-up of PEL		X (50%)	X	X
Outpatient follow-up should be from 4 to 8 weeks	X (83.3%)		X	X

ENT, otolaryngologist; PED, pediatrician; PEL, post extubation laryngitis; FNL, Flexible Nasolaryngoscopy; PICU, Pediatric Intensive Care Unit; NICU, Neonatal Intensive Care Unit; GERD, Gastroesophageal Reflux Disease; PPI, proton pump inhibitors; NIV, non-invasive ventilation; MLB, microlaryngoscopy; SGS, subglottic stenosis.

## Discussion

Inflammation of the larynx related to tracheal intubation is referred to by a number of different names in the literature, such as post-intubation laryngeal injuries, post-extubation stridor, post-intubation laryngitis, intubation lesions, post-intubation acute laryngeal injuries. The consensus nomenclature considered most appropriate was post-extubation laryngitis (PEL). Despite the different names given to this situation, all participants agreed that the diagnosis refers to an acquired stridor and/or dysphonia and/or effortful breathing beginning hours or days following extubation that may be associated to failed extubation or not, and that a definitive diagnosis of laryngitis is only established through endoscopic confirmation of characteristic laryngeal lesions.

In the current expert panel, stridor was considered the most important symptom by most study members. Cordeiro et al., in their prospective study, investigated the effectiveness of clinical evaluation in identifying moderate or severe airway injuries resulting from endotracheal intubation in children when compared to airway endoscopy.^5^ The authors utilized the Downes and Raphaelly score^6^ and found that clinical evaluation was valuable in excluding moderate and severe airway injuries, although a low score demonstrated low specificity. In a recently published prospective cohort study, the accuracy of stridor in diagnosing pediatric post-intubation subglottic stenosis was examined. Stridor exhibited high specificity in detecting subglottic stenosis, particularly when it persisted for over 72 h or started more than 72 h after extubation. Conversely, the absence of stridor was sufficient to rule out post-extubation subglottic stenosis.^7^ This is particularly relevant to pediatricians who do not work in close contact with airway endoscopists to identify patients that may need further investigation even after discharge. Stridor initiated after intubation, despite length of intubation, should alert to the presence of post-extubation laryngeal lesions and airway obstruction. Nevertheless, in this expert consensus, a multidisciplinary team with routine endoscopic evaluation was considered the optimal approach to accurately diagnose PEL.

Regarding length of intubation, laryngeal lesions may manifest even after a brief period of endotracheal intubation, especially in cases of traumatic and challenging intubations.^8^ There was no consensus on the secure upper limit for the duration of intubation in children, which is in accordance to Principi et al.^9^ Nevertheless, a study addressing length of intubation as a risk factor for SGS showed in a multivariate analysis that for every five additional days of intubation there was a 50.3% increase in the risk of developing SGS.^10^ Length of intubation emerges as an independent risk factor for SGS, even though the existing literature does not establish an upper safe limit for the duration of intubation.

In this consensus, previous history of intubation and Gastroesophageal Reflux Disease (GERD) are considered risk factors for PEL. Predisposing factors for PEL in literature also include oversized endotracheal tube, presence of systemic diseases, underdiagnosed congenital airway narrowing, traumatic nasogastric or endotracheal tube suctions, as well as faulty intubation techniques (traumatic and multiple intubations) in an insufficiently anesthetized patient with excessive tube motion.^10^, ^11^ Although infants are more tolerant to intubation than older children, some reports have suggested that preterm infants are more prone than older children to develop this condition.^12^

Endoscopic assessment may determine the degree of acute post-intubation injuries and support the decision of the best treatment options and plans for elective extubation. In this consensus, most agreed that 24 h–48 h is the maximum time one should expect for spontaneous resolution of symptoms before demanding an endoscopic evaluation. Obstructive symptoms that improve in 24 h–48 h are probably caused by mild lesions limited to mucosal edema.^7^

In agreement with Schweiger et al.,^13^ consensus was achieved regarding which lesions were considered the most severe form of PEL: circumferential mucosal ulceration, ulceration with exposed cartilage, SGS and interarytenoid lesions. As introduced in the same study, the CALI classification has proven to be particularly useful and practical. Knowledge of the types of lesions and visual footage of endoscopic aspects have contributed to a better medical treatment decision on a case-to-case discussion.^13^

The importance of clinical and endoscopic follow-up cannot be overstressed. The scarring process of acute laryngeal lesions may be quite entirely unexpected since individual inflammatory reactions and scarring processes are variable and not completely understood. Clinical practice and observation have shown that, most of the time, mild lesions can be successfully treated with clinical measures, whereas moderate and severe lesions most probably will need endoscopic surgical treatment.

Medical treatment modalities include systemic and nebulized medications, laryngeal rest, and ventilatory support. Surgical modalities include removal of granulation tissue, dilation, and endoscopic injection or topical application of medications.^11^ The rationale for these treatments is to minimize exuberant and noticeably obstructive inflammatory reactions, to maintain a patent airway, and to promote reepithelization of ulcerations.^11^

The use of systemic corticosteroids as part of the clinical treatment was a consensus if there is no contra-indication. Dexamethasone is the most frequently used medication. Minimal clinical treatment length with systemic corticosteroid was defined as 48 h. There was, however, no agreement on the minimum and maximum dose of dexamethasone: 47.1% use 0.6 mg/kg/day, 29.4% less, and 23.5% more than 0.6 mg/kg/day. When inquired about the maximum dosage of prednisolone, there was no consensus: 41.2% said this to be less than 1 mg/kg/day, 23.5% use up to 1 mg/kg/day, and 35.3% would use up to 2 mg/kg/day. Nebulized adrenaline is considered part of clinical treatment.

Although scientific evidence currently fails to support inhaled epinephrine and corticosteroids to treat PEL, some studies and a 2009 Cochrane review have suggested a trend toward benefit.^14^, ^15^, ^16^

Proton pump inhibitors (PPI) are used as treatment and dosage varies from 1 to 2 mg/kg/day. The use of PPI is supported by animal studies showing that subglottic wound healing is significantly affected by pepsin and bile acid under acidic conditions and not under non-acidic conditions. This would justify the use of acid-suppression therapy when there are glottic/subglottic lesions to prevent or reduce inflammation and subglottic stenosis.^17^ This concept is widely known amongst airway surgeons following open airway surgery.^18^ In preterm infants, nevertheless, use of proton pump inhibitors is significantly associated to gram-negative sepsis and necrotizing enterocolitis^19^, ^20^ and therefore its use in neonates should be limited to high suspicion of reflux and consideration taken to risks versus benefits on a case-to-case basis.

Noninvasive Ventilation (NIV) is considered beneficial and useful (100%) and this has been found in literature as the sole modality shown to have sufficient evidence to support its use.^21^ Inadequate sedation levels, inappropriate masks, craniofacial deformities, nasal lesions and inexperienced physiotherapy teams are considered the causes for NIV failure reaching high consensus in the group. High flow nasal cannula can also be a useful tool in treating PEL in expert’s view.

“Laryngeal rest”, translated as adequate sedation levels and minimum manipulation of the larynx, is considered important. Experts consider the use of Comfort scales beneficial. Atraumatic pharyngeal or endotracheal tube suctions are also critical in minimizing mucosal lesions, and proper suspension of the tube connector to the ventilator’s tube diminishes the risk of tube motion and, therefore, laryngeal lesions.^11^

When symptoms (stridor and dysphonia) continue or do not improve over the first 72 h after extubation and clinical treatment, a microlaryngoscopy (MLB) under general anesthesia should be performed. This is also when second extubation fails, and severe acute laryngeal lesions are seen on Flexible Nasolaryngoscopy (FNL).

**Figure 1:**
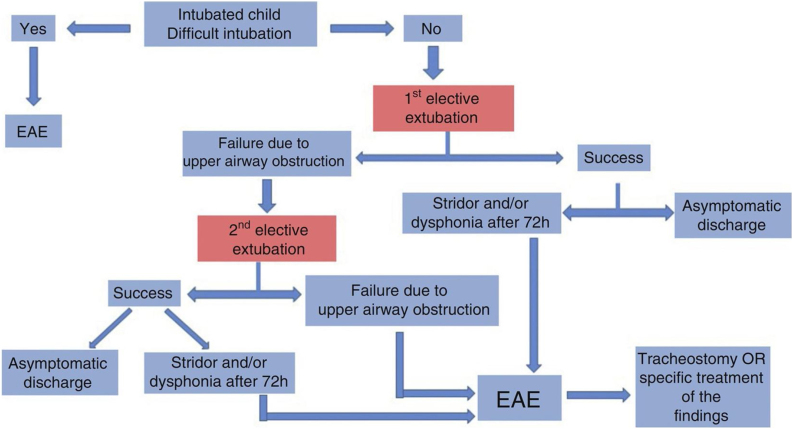
Flowchart indicating the need of endoscopic airway evaluation in the intubated child. Figure authorized by the authors.^22^

An algorithm (Fig. 1) has been suggested in the First Brazilian Clinical Consensus on Tracheostomized Children to help define the indication for Endoscopic Airway Evaluation (EAE) or MLB.^22^

MLB should be performed using a 0° or 30° telescope under general anesthesia and spontaneous ventilation. This allows for differential diagnosis with laryngomalacia, tracheomalacia, and vocal cord palsy, although the latter should be confirmed with an awake endoscopy.^11^ Airway calibration with an endotracheal tube is routine during MLB.

When dilation is indicated for acute SGS, using a balloon is currently the method of choice. Acute laryngeal stenosis is a time-sensitive lesion with success rates reported as high as 100%, varying greatly for chronic cases.^23^ This fact justifies treatment in the first weeks following extubation, attempting to prevent the need for tracheostomies and for future open airway surgery.

Characteristics of laryngeal lesions will define endoscopic measures that are chosen, following principles of potentially removing obstructive granulation tissue, debris and fibrin from ulcerations and treating inflammatory mucosa with corticosteroid.^11^ Triamcinolone is the most frequently used corticosteroid for intra or perilesional infiltration.

In children who have experienced extubation failure before endoscopic treatment, after removing obstruction and cleansing of airway lesions, a one or half-size smaller uncuffed tube is employed for reintubation. Extubation is planned after 24–72 h of further clinical treatment. Antibiotic and corticosteroid ointment applied around the tube is used by all the otolaryngologists participating in this consensus.

Endoscopic findings and prior use of systemic corticosteroids will determine the need for further use of systemic dexamethasone. Taking cultures of tracheal secretion is not part of the routine. It is indicated when secondary infection is suspected, in cases of poor outcome, and/or severe lesions are encountered in endoscopy.

At this active larynx stage, performing a tracheostomy may worsen the laryngeal condition.^11^ Infected granulations evolve over time into contracting scars, leading to severe cicatricial sequelae.^11^ If a tracheotomy cannot be avoided despite adequate endoscopic treatment, proper tracheostomy tube placement^18^ and follow-up are crucial. The ENT surgeon is responsible for the tracheostomy indication and should be involved in the follow-up. This is obviously biased considering selection of experts involved in the multidisciplinary teams where the otolaryngologists were routinely involved.

The need of MLB in follow-up in not consensus, but ENT should perform reevaluation in outpatient setting after 1–3 weeks regardless of symptoms, and outpatient follow-up should be from 4 to 8 weeks.

It is important to highlight that evidence-based practice for pediatric PEL is notably limited. Consequently, it underscores the significance of multidisciplinary team discussions, on a case-by-case basis, for best decisions regarding corticosteroid dosages, antibiotic and PPI treatments, and timing for tracheostomy to manage persistent laryngeal obstruction.

## Conclusion

This study provides guidance to manage young infants with PEL, which is the recommended term used for acute laryngeal lesions caused by intubation. Diagnosis should be confirmed by airway endoscopic evaluation, with stridor as a key symptom. There is no consensus on safe intubation length. Systemic corticosteroids (usually dexamethasone), nebulized adrenaline, and proton pump inhibitors are options for clinical management. Noninvasive ventilation and laryngeal rest are also vital. If symptoms persist or worsen within the first 72 h after extubation despite maximum clinical treatment or in the case of a second extubation failure, microlaryngoscopy under anesthesia is recommended. This is particularly important when findings from Flexible Nasolaryngoscopy are inconclusive or when moderate to severe laryngeal lesions are identified.

## Authors’ contributions

All authors contributed to the conception of the study and acquisition of data. Débora Bressan Pazinatto, Rebecca Maunsell, Melissa Ameloti Gomes Avelino were responsible for analysis of organization of results and drafting of the original version. Jose Faibes Lubianca Neto, Cláudia Schweiger and Jamil Pedro de Siqueira Caldas contributed to interpretation of data, drafting the article and final approval of the submitted version. Carlos Takahiro Chone contributed to revision of submitted version.

## Conflicts of interest

The authors declare no conflicts of interest.
